# The good rays: let them shine!

**DOI:** 10.1007/s00259-018-4233-7

**Published:** 2018-12-17

**Authors:** Poul F. Høilund-Carlsen

**Affiliations:** 1 Department of Nuclear Medicine, Odense University Hospital, 5000 Odense C, Denmark; 2 Department of Clinical Research, Faculty of Health Sciences, University of Southern Denmark, Odense, Denmark

Nobody will deny that high-dose ionizing radiation known from nuclear bombs and power plant catastrophes is extremely dangerous and something that we need to guard against with strict security measures. Nor will anybody contest the need for rules for low-dose ionizing radiation (LDR) in medical use to safeguard staff and patients from excessive exposure, not to mention healthy control subjects needed for comparison in research studies. It seems, however, that we have gone too far in protection against LDR, thereby cutting ourselves off from gaining crucial new knowledge, improving diagnostics and achieving breakthroughs in the management of serious diseases, in which refined medical imaging employing LDR would no doubt make a substantial difference.

Current rules and limitations on the use of medical LDR are based on a hypothetical model, the linear no threshold (LNT) concept, which has never been proved to be right. The result is too tight regulations that limit the development and use of molecular imaging and prevent its potential from being fully unfolded to the benefit of patients and society. Since this serves no-one’s interest, the rules need to be changed to facilitate and not complicate the realization of this potential. This excessive restriction is particularly regrettable considering that molecular imaging is about to revolutionize our perception of many of the worst diseases that afflict mankind and to significantly improve their management.

Here we argue that LDR is widely inert and should be used for medical imaging more extensively and without restrictions as long as the effective dose to the patient from a single exposure or the annual cumulated dose from repeat examination stays below 100 mSv, or even 200 mSv. It is time that the authorities and regulatory boards acknowledge this and act accordingly instead of being stuck with a hypothetical model that has little to do with reality, and the validity of which has been consistently disproved. This necessary change will mean a goodbye to the LNT model and a long-needed relaxation of the current regulations with their excessively low arbitrary dose limits which, unfortunately, are still recognized by the scientific ethics committees. To trigger this highly required change is the main purpose of this editorial.

**Fig. 1:**
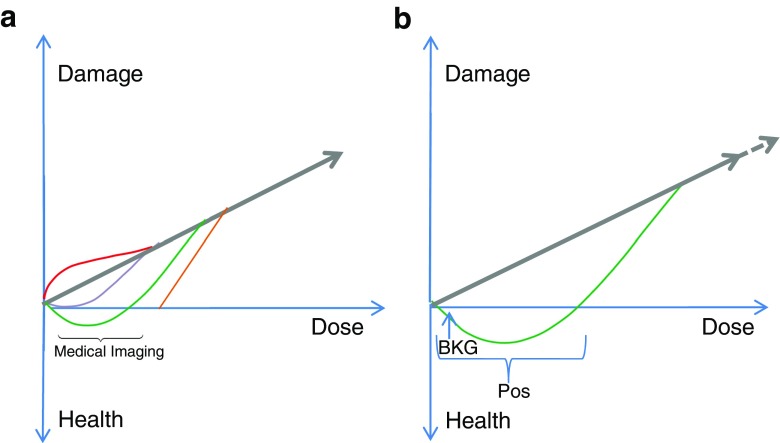
**a** Linear no threshold hypothesis. The straight line illustrates the LNT concept that any radiation dose, no matter how small, causes damage, and that damage increases linearly with dose. The coloured lines indicate other theories: *red* damage increases, *lilac* damage decreases, *green* radiation has a beneficial effect, *orange* threshold hypothesis. **b** Biphasic dose response (enlarged view of the green curve in **a**). The horizontal brackets indicates the interval of positive LDR effects (*Pos*), while doses above are increasingly damaging. Whether deficiency symptoms may appear due to insufficient radiation is unknown, since all people are exposed to a natural background radiation (*BKG*) of some size

The misfortune of radiation regulation was the introduction of the LNT concept about 70 years ago. The concept is based on the opinions of the American biologist and Nobel Laureate Hermann Joseph Muller, i.e. it is a purely theoretical model, stating that tissue damage increases linearly with the radiation dose, and that any radiation, no matter how small the dose, causes damage (Fig. 1a) [1]. Confronting this concept is the view that LDR is harmless and within certain dose ranges perhaps even beneficial and desirable [2]. This is a perception that with time is being shared by many professionals, since a multitude of observations accumulated during the last 100 years have, piece by piece and almost unanimously, contradicted the LNT concept, which, however, the authorities have chosen to disregard for decades. A biphasic response curve (Fig. 1b) is what toxicologist Edward J. Calabrese and coworkers from the study of thousands of biological systems consider nature’s ‘law’ rather than a linear association, meaning that an agent at low doses may stimulate, while at higher doses will increasingly inhibit or damage, whereas a linear relationship, like the one postulated by the LNT hypothesis, is hardly ever encountered in the biological setting [3, 4].

**Fig. 2:**
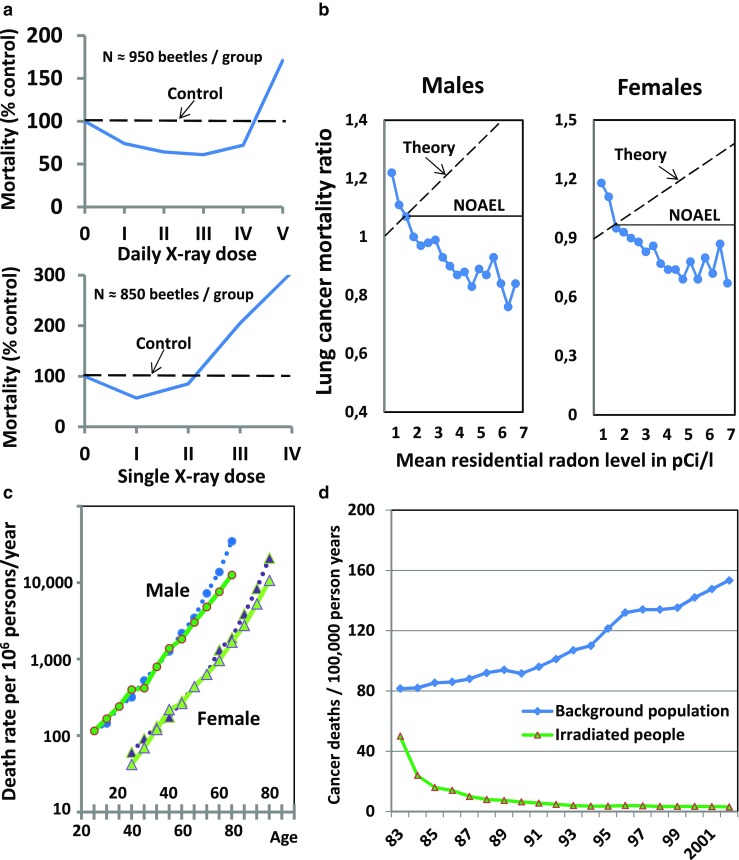
Examples contradicting the LNT hypothesis selected from the literature from the last 100 years. **a** Prolonged life of flour beetles after multiple daily low doses (*upper panel*) or a single low dose (*lower panel*) of X-rays compared to nonirradiated controls (*dashed horizontal curves*) Modified from [5]. **b** Observed decreases (opposite to theory) in lung cancer mortality rates (corrected for smoking) with increasing residential exposure to radon in 1,601 (90% of all) US counties (*NOAEL* no adverse effect level). From data reported in [9]. **c** Increased life time (*solid curves*) of Nagasaki A-bomb survivors exposed to low dose radiation compared to nonexposed controls (*dotted curves*) [8]. **d** Mortality of 10,000 occupants of 1,700 ^60^Co-contaminated apartments in Taiwan who received a mean yearly dose of 49 mSv or an accumulated dose of 400 mSv during their total stay (*lower curve*) versus mortality in the general population (*upper curve*). In the exposed population, the rate of congenital malformations was only 7% of that in the general population [10]

Long before the advent of the LNT model, the first studies contradicting it had appeared. In an elegant study, Davey convincingly demonstrated in 1919 that small doses of X-rays apparently prolonged the life of the flour beetle *Tribolium confusum* (Fig. 2a) [5]. Since then, as pointed out by highly experienced professionals including Ludwig E. Feinendegen and Myron Pollycove, “all statistically significant, adequately controlled epidemiological studies confirm LDR is associated with reduced mortality from all causes, decreased cancer mortality, and may be protective against high-dose radiation” [6, 7]. The examples shown in Fig. 2 show excess longevity of Nagasaki A-bomb survivors exposed to estimated low radiation doses (Fig. 2b) [8], decreases in lung cancer mortality rates with increasing radon exposure (Fig. 2c) [9], and many fewer cancer deaths (and fewer congenital malformations) among inhabitants of ^60^Co-contaminated apartments receiving a yearly dose of about 50 mSv than in the background population (Fig. 2d) [10].

These and multiple other, often unexpected but scientifically valid, findings have not made authoritative boards change their regulations, although they are supposed to keep a keen eye on the literature and react when current rules are in need of adjustment. Authors have made controversial results go away, for instance, by setting all estimated doses below 50 mSv equal to zero or pooling the results from exposure to LDR with those from exposure to much higher doses, as done in investigations of Hiroshima and Nagasaki survivors [11]. Misleading examples have continued to appear. Thus, in the 100-year (1897–1997) study presenting rates of death and cancer among British radiologists, the authors report “an increasing trend in risk of cancer mortality with time since first registration with a radiological society (p=0.002), such that in those registered for more than 40 years there was a 41% excess risk of cancer mortality” [12]. This was completely repudiated in a commentary by American hospital physicist John R. Cameron, who used the authors’ data to demonstrate that even the earliest radiologists did not suffer a decrease in longevity despite large exposures (estimated >1 Sv year^−1^) before 1920 when the first radiological protection recommendations appeared, and that the later radiologists showed no difference in cancer rates compared with other physicians, but significantly lower cancer rates than all men in England and Wales and all social class I peers. Furthermore, since 1936, cancer rates among radiologists dropped below those in the general public, and radiologists registered after 1955 had a 32% lower (*p* < 0.001) mortality rate for all-cause deaths than that of all physicians, a 36% lower (*p* < 0.001) mortality rate for noncancer deaths than other physicians, and a 29% lower (not significant) mortality rate for cancer than that of all male physicians, and these data relate to a period during most of which the exposure limit for occupational workers was 50 mSv year^−1^ [13].

Even more misleading are the results of studies indicating excess cancer mortalities in such large populations of nuclear industry workers that the conclusions are easily accepted at face value, while a closer inspection reveals that the estimates were obtained simply by multiplying the number of workers receiving a particular radiation dose with an undefined relative risk applying the LNT model [14], instead of comparing the cancer rates with those in appropriate control populations. The fear generated in people and communities by results obtained by this kind of circular reasoning not only creates serious negative consequences, but also “huge expenditures to avoid radiation exposure even at low doses at which detrimental effects are not observed” [15]. Diligent correction of one such study revealed by comparison with a suitable background population that the all-cause mortality ratio in nuclear workers was 0.76 compared with 1.02 in non-nuclear workers, a difference of 16 standard deviations, and that the ratios for death due to all malignant neoplasms were 0.95 and 1.12 (*p* < 0.001), respectively, a difference of four standard deviations [16].

Authoritative boards such as the United Nations Scientific Committee on the Effects of Atomic Radiation (UNSCEAR) and the National Academy of Sciences (NAS) in its Biological Effects of Ionizing Radiation (BEIR) reports have been well aware of the difficulties in acquiring “reliable information about the correlation between small doses and their effects either in individuals or in large populations”, but have consistently sought to minimize the significance of this by statements such as the following in BEIR VII from 2006: “the committee concludes that the preponderance of information indicates that there will be some risk, even at low doses” [17].

**Fig. 3:**
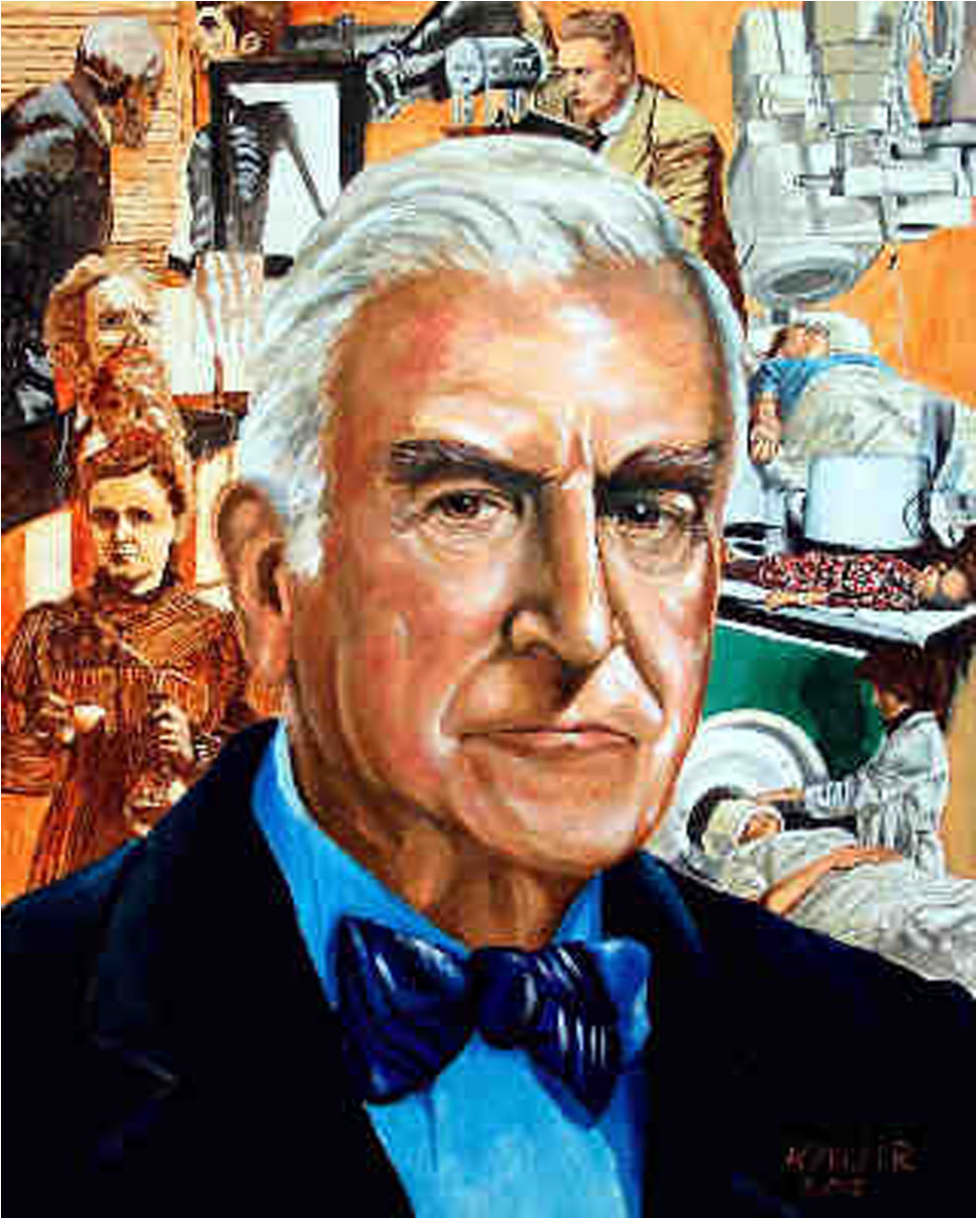
Lauriston S. Taylor (1902–2004), founder of the ICRP and NCRP, past editor-in-chief of *Operational Radiation Safety* and of *Health Physics* (painting by Kenneth L. Miller)

The debate has been lengthy. Many of those who more than 80 years ago established the first regulations in the field became very old, and as time went by came to the conclusion that LDR is not harmful. A prominent example is Lauriston S. Taylor, founder of the International Commission on Radiation Protection and the National Council on Radiation Protection and Measurements in the US (Fig. 3). He was 102 when he died and at the age of 89 made the following statement: “No one has been identifiable injured by radiation while working within the first numerical standards set first by the NCRP and then by the ICRP in 1934 (0.2 R day^−1^ or approx. 500 mSv year^−1^)”. And “The theories about people being injured have still not led to demonstration of injury and, though considered as facts by some, must only be looked upon as figments of the imagination” [18]. The same attitude is now expressed by the International Organization for Medical Physics, that represents 80 national and six regional medical physics organizations and 18,000 medical physicists worldwide, as described by the organization head William R. Hendee [19]. Similarly, the American Association of Physicists in Medicine writes in its position statement of April 2018 [20]:

At the present time, epidemiological evidence supporting increased cancer incidence or mortality from radiation doses below 100 mSv is inconclusive. As diagnostic imaging doses are typically much lower than 100 mSv, when such exposures are medically appropriate, the anticipated benefits to the patient are highly likely to outweigh any small potential risks.

Given the lack of scientific consensus about potential risks from low doses of radiation, predictions of hypothetical cancer incidence and mortality from the use of diagnostic imaging are highly speculative. The AAPM, and other radiation protection organizations, specifically discourages these predictions of hypothetical harm. Such predictions can lead to sensationalistic stories in the public media. This may lead some patients to fear or refuse safe and appropriate medical imaging, to the detriment of the patient.

The current regulations have done well for many years, so why slacken them? The answer is simple. X-rays and radioactivity were discovered more than 120 years ago, and yet their potential in medical imaging has still not been fully exploited while the cost of healthcare is on a steep rise and more individualized diagnostics and therapy are called for. An example of this is PET imaging with ^18^F-NaF to detect and grade early arterial wall microcalcification when it may still be amenable to therapy [21]. NaF imaging is a methodology that may potentially lead to a breakthrough in the management of atherosclerosis, the world’s number one killing disease [22], which may afflict us all if we grow old enough. However, to achieve this, longitudinal studies with NaF PET/CT, sometimes in combination with FDG PET/CT, are absolutely necessary. Nonetheless, to our knowledge, our Ethics Committee has not allowed the number of repeat examinations that are necessary to study disease progression in a prospective design. This is a serious obstacle to utilizing an important method without any risk from repetition, because the accumulated radiation dose amounts to only about 40 mSv distributed over several (5 or 6) years. Referring to current guidelines for the use of ionizing radiation in medical research, the Ethics Committee requires particularly strong arguments for the use of such doses in patients and finds them unacceptable in healthy control subjects. Therefore, one may ask who is favoured by the current tight radiation limits, when multiple observations confirm that annual radiation doses up to 100 mSv or more are completely harmless [23, 24]. According to a recent article by Siegel et al. in the *Journal of Nuclear Medicine*: “the ‘prudence’ of dose optimization … is responsible for misguided concerns promoting radiophobia, leading to actual risks far greater than the hypothetical carcinogenic risk purportedly avoided” [25].

Two quotations appear if one starts considering how a concept that is so obviously wrong can survive for so many years. One is from Goethe’s play *Torquato Tasso*: “*So fühlt man Absicht, und man ist verstimmt*” (“One sees the intention and gets depressed”). The other is the more commonly known Latin quotation: “*Errare humanum est*” (“To err is human”), the continuation of which, however, “(*sed*) *perseverare diabolicum*” (“(but) to persist is diabolical”) is often forgotten. Which quotation fits the actual situation better is a matter of choice. What is not debatable, however, is that the continuation should always be kept in mind. Thus, it is time that the current stifling restrictions are greatly relaxed, to the benefit not only of patients, but also of society in general.
